# Neoadjuvant chemotherapy in technically unresectable head and neck cancers: a retrospective audit

**DOI:** 10.3332/ecancer.2022.1460

**Published:** 2022-11-02

**Authors:** Bal Krishna Mishra, Akhil Kapoor, Anuj Gupta, Bipinesh Sansar, Arpita Singh, Somnath Roy, Tanmoy Mandal, Sujay Srinivas, Sudeep Das, Aseem Mishra, Ashutosh Mukherjee, Sambit Nanda, Kurupathy Sambasivaiah

**Affiliations:** 1 Department of Medical Oncology, Mahamana Pandit Madan Mohan Malviya Cancer Centre & Homi Bhabha Cancer Hospital, Tata Memorial Centre, Varanasi, 221005, India; 2 Department of Head and Neck Surgery, Mahamana Pandit Madan Mohan Malviya Cancer Centre & Homi Bhabha Cancer Hospital, Tata Memorial Centre, Varanasi, 221005, India; 3 Department of Radiation Oncology, Mahamana Pandit Madan Mohan Malviya Cancer Centre & Homi Bhabha Cancer Hospital, Tata Memorial Centre, Varanasi, 221005, India

**Keywords:** neoadjuvant chemotherapy, technically unresectable, head and neck cancers

## Abstract

**Background:**

The data regarding the use of neoadjuvant chemotherapy in technically unresectable head and neck cancer (HNC) is limited and real-world studies are needed to look for the efficacy and toxicities of this approach.

**Patients and methods:**

This is a retrospective study conducted in the Medical Oncology department of our hospital. All technically unresectable HNC patients who underwent neoadjuvant chemotherapy between May 2018 and May 2020 were included in this analysis. Patients received three-drug regimen docetaxel, cisplatin and 5-fluorouracil (DCF) regimen, two-drug regimens included docetaxel + cisplatin, paclitaxel + carboplatin both weekly and 3-weekly. The resectability assessment was done clinically and radiologically after completing three neoadjuvant cycles. Overall survival was calculated from the first day of chemotherapy to the date of last follow-up or date of death.

**Results:**

A total of 119 patients received neoadjuvant chemotherapy during the specified time. Response assessment showed partial response in 41.9% of patients with three-drug regimens and 37.5% of patients with other regimens. Out of 119 patients, 56 (47%) patients were offered radical intent therapy. Resectability was achieved in 32.3% of three-drug regimen patients and 26.1% of other patients. Surgery was feasible in 33 (27.7%) patients, and postoperative radiotherapy and concurrent chemotherapy were done in 30 patients (25.2%), and surgery with only postoperative radiotherapy was done in 3 patients (2.5%). Radical chemoradiotherapy was done in 23 patients (19.3%). The estimated median survival for patients who could undergo surgery was 18 months [95% confidence interval (CI), 14.9-21.0], and nonsurgical patients were 9 months (95% CI, 7.3–10.6) (p = 0.0001).

**Conclusion:**

Our study shows that neoadjuvant chemotherapy in technically unresectable HNC patients can make the disease resectable in around one-third of the patients. The patients who could undergo surgery after neoadjuvant chemotherapy had significantly improved survival as compared to those who could not.

## Introduction

Head and neck cancer (HNC) is a significant health problem in India due to tobacco chewing, especially in the male population [1]. Another reality is their presentation as a locally advanced disease which ranges from 58.3% to 76.9% for various subsites in various parts of countries [2].

Surgery is the primary treatment for oral cavity cancer [3–8]. Advanced stage HNC is mainly treated by combined modality treatment, in which surgery followed by radiotherapy with concurrent chemotherapy is delivered. However, in oral cavity cancers, if surgery is not technically feasible, the treatment is essentially palliative. Concurrent chemoradiation has been tried in these patients; however, the outcomes are dismal with a median progression-free survival (PFS) of 6.4 months. Besides, it is challenging to get a clear margin in locally advanced HNC. The local recurrence rate is higher in patients with close or positive margins than negative margin patients [9]. Thus, it is necessary to get surgical resection with negative margins [10]. Decisions regarding resectability and intent of the treatment in borderline cases should always be made by a multidisciplinary team. There is already some data to suggest that a proportion of carefully selected patients with borderline unresectable tumours who would not be offered surgery might be made resectable using induction chemotherapy. TAX 323 and 324 are regarded as landmark studies that have used neoadjuvant chemotherapy in locally advanced HNC. The response rates with the three-drug regime were to the tune of 68%; however oral cavity cancers constituted a minority of the treated patient population in these studies. The data regarding the same is conflicting and real-world studies are needed to look for the efficacy and toxicities of this approach.

## Materials and methods

### General study details

This is a retrospective study conducted in the Medical Oncology department of our hospital. All technically unresectable HNC patients who underwent neoadjuvant chemotherapy (NACT) between May 2018 and May 2020 were included in this analysis. Since this is an audit of the standard institutional practice, ethical approval was not sought. The study was conducted according to ethical guidelines established by the Declaration of Helsinki and other guidelines like Good Clinical Practice Guidelines and those established by the Indian Council of Medical Research.

All HNC patients were evaluated by a multidisciplinary clinic, including surgical oncologists, radiation oncologists, medical oncologists and radiologists. All technically unresectable patients were planned for neoadjuvant chemotherapy treatment. The reasons for labeling the disease as technically unresectable were as follows:

1. Disease reaching up to zygoma and/or soft tissue swelling up to zygoma
2. Infratemporal fossa involvement
3. Extensive soft tissue involvement reaching up to the hyoid cartilage and extensive skin infiltration
4. Fix lymph nodes with vascular involvement.

All technically unresectable patients received neoadjuvant chemotherapy with various regimens, which includes a three-drug regimen DCF (docetaxel + cisplatin + 5-fluorouracil), two-drug regimens include DC (docetaxel + cisplatin), PC (paclitaxel + carboplatin) both weekly and 3- weekly. The number of cycles given was three in 3-weekly regimens and eight in weekly PC regimens. Docetaxel was administered at dose of 75 mg/m^2^ over 1 hour on day 1, cisplatin at 75 mg/m^2^ over 1 hour divided in 2 days, 5-fluorouracil at dose of 750 mg/m^2^ continuous infusion over 24 hours day 1–5 via a peripherally inserted central catheter. Paclitaxel was given at dose of 175 mg/m^2^ on day 1 and carboplatin at Area under curve (AUC) 5 for 3-weekly protocol, while the dose was 80 mg/m^2^ and AUC2, respectively, for weekly protocol. Patients having grade ¾ toxicities were considered for next cycle of chemotherapy at 80% doses.

Toxicity data were collected mainly for anaemia, febrile neutropenia, thrombocytopenia, vomiting, diarrhoea and cardiac toxicity to see the feasibility of all regimens as per Common Terminology Criteria for Adverse Events (CTCAE) version 4.02. All standard DCF regimen patients received prophylactic G-CSF. All patients treated with other regimens also received granulocyte colony stimulating factor (G-CSF). if they developed febrile neutropenia in the previous cycle.

The resectability assessment was done clinically and radiologically after completing three neoadjuvant cycles. All patients were assessed for resectability in the multidisciplinary joint clinic. All patients who were not found resectable were planned for radical chemoradiation (CT RT), radical radiotherapy, palliative radiotherapy, palliative chemotherapy and best supportive care as per joint clinic decision.

We looked at age, sex, site, histopathology, the indication of neoadjuvant chemotherapy, number of chemotherapy cycles, type of chemotherapy regimen, type of treatment given after completion of neoadjuvant therapy, response and survival for all patients.

*Statistics*

We did statistical analysis by using Statistical Package for Social Sciences (SPSS) software version 16. We calculated overall survival (OS) from the first day of chemotherapy to the date of last follow-up or date of death.

**Results**

*Baseline parameters*

A total of 119 patients received neoadjuvant chemotherapy during the specified time period. Median age, male/female ratio, site, reason of neoadjuvant chemotherapy and type of chemotherapy regimen are reported in Table 1. 26.2% (*n* = 31) of patients received three drug-based regimens in comparison to 73.8% (*n* = 88) of patients who received two-drug regimen. In the three-drug regimen, standard DCF regimen was used. Two-drug regimens include DC, PC both weekly and 3-weekly. The cycles given were three in 3-weekly regimens and eight in weekly PC regimens. Out of 88 patients receiving doublet chemotherapy regimen, 42 (47.7%) received DC protocol, 30 (34.1%) received 3-weekly PC and 16 (18.2%) received weekly PC regimen.

### Efficacy

Response assessment showed partial response (PR) in 41.9% of patients with three-drug regimens and 37.5% of patients with other regimens (Table 2). Complete response (CR) was not seen with any regimen. Resectability was achieved in 32.3% of three-drug regimen patients and 26.1% of other patients. Table 3 shows the responses as per the indication of NACT. Among all the indications of NACT, partial responses were highest for disease/oedema up to zygoma.

### Toxicity

Grade 3 and 4 toxicities were reported with all types of regimens, and the main toxicities seen were anaemia, febrile neutropenia, thrombocytopenia, vomiting, diarrhoea and cardiac (Table 2). No death was reported because of toxicity, and except for anaemia which was more common in two-drug arm, none of the toxicity showed a significant difference in both arms.

### Post-neoadjuvant therapy treatment

Out of 119 patients, 56 (47%) patients were offered radical intent therapy (Table 4). Surgery was feasible in 33 (27.7%) patients, and postoperative radiotherapy and concurrent chemotherapy were done in 30 patients (25.2%), and surgery with only postoperative radiotherapy was done in 3 patients (2.5%). Radical chemoradiotherapy was done in 23 patients (19.3%). Sixty three patients (53%) were not fit for any radical treatment, and 48 patients (40.4%) received palliative chemotherapy, 8 patients (6.7%) received palliative radiotherapy and 7 patients (5.9%) received only best supportive care. The dose of radiation used was 66–70 Gy/6–7 weeks with a fraction size of 2 Gy for radical radiotherapy and 60 Gy/6 weeks for postoperative radiotherapy with a fraction size of 2 Gy.

### Overall survival

**Figure 1.**
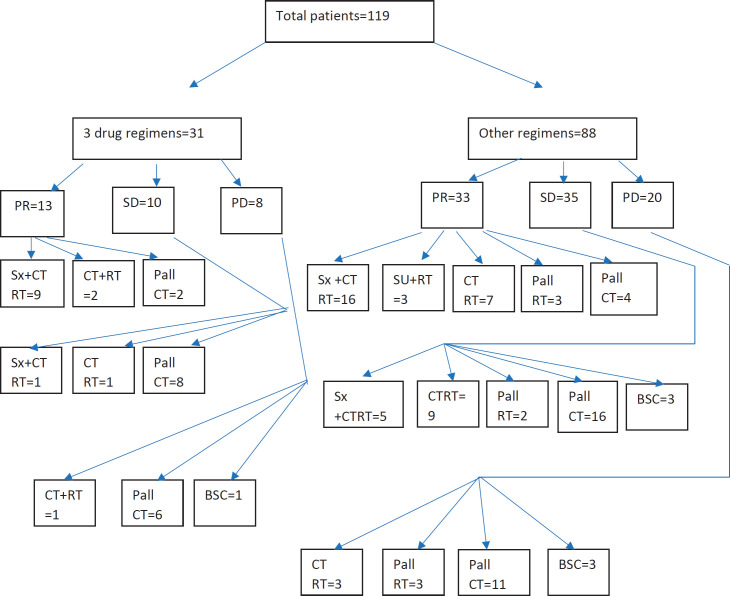
Consort diagram of all patients. CR, Complete response; PR, Partial response; SD, Stable disease; PD, Progressive disease; Sx, Surgery; CTRT, Chemoradiation; Pall RT, Palliative radiation; Pall CT, Palliative chemotherapy; BSC, Best supportive care.

The median duration of follow-up was 27 months (95% CI, 15.1–38.9). Total 85 events had taken place during this time. The estimated median OS for the whole population is 12 months (95% CI, 10.1–13.9). The estimated median survival for surgical patients was 18 months (95% CI, 14.9–21.0) and nonsurgical patients were 9 months (95% CI, 7.3–10.6), and this was statistically significant (*p* = 0.0001) (Figure 1).

## Discussion

Oral cavity cancers have a high prevalence in South-Central Asian men, with most of them presenting as locoregionally advanced disease. The advanced stage at presentation always remains a challenge to plan curative-intent treatment. Surgery for local and regional disease combined with adjuvant therapy, even in cases of advanced disease, can offer a very reasonable survival rate [18]. Various neoadjuvant chemotherapy trials in HNCs did not show any benefits [20, 21]. Patil *et al* [16] reported the data of 123 patients and demonstrated the effectiveness of induction chemotherapy in down-staging of tumours. He reported median OS for the whole population of 12.7 months, and at the same time, estimated median survival was not reached for patients who had undergone post-chemotherapy resection. The difference was statistically significant (*p* = 0.0001). In another study by Patil *et al* [19], 721 technically unresectable patients who received neoadjuvant chemotherapy were retrospectively analysed. 43% of patients able to undergo surgery and median OS was 19.6 months who received surgical treatment compared to 8.16 months in those patients in which surgery was not possible, contrary to other neoadjuvant chemotherapy trials. It was observed that the proportion of oral cavity cancers in all major trials conducted primarily in North America and Europe was around 14%–18%, and maybe this low percentage may be the reason for different results.

The issue of borderline resectability is much more frequent in oral cancer, and it may need a different approach for management. We offered neoadjuvant chemotherapy to only those technically unresectable patients having features mentioned in the method section.

NACT protocol used in our study was based on published literature documenting the efficacy of both three drugs, two drugs and other regimens [22, 23]. In our analysis, 26.2% of patients received three-drug regimens, and 73.8% received two-drug regimens or other regimens and were found to be efficacious. The PR rates were 41.9% with three-drug regimen and 37.5% with two-drug regimens, which are actually lower than those reported in other studies [24, 25]. The low response may be due to more advanced stage patients and more oral cavity patients in our cohort, which is traditionally associated with poor response [22, 25].

32.3% of patients in the three-drug arm versus 26% in another arm could undergo surgery in our study with R0 resection, which was a significant achievement. Median OS was 18 months in patients who had surgical resection with adjuvant chemoradiation or adjuvant radiation compared to 9 months who could not go for surgical resection and were treated with radical chemoradiation, radical chemoradiation, radical radiation and other therapies (*p* = 0.0001). It shows the positive impact of surgery on survival. This was in concordance with EORTC postoperative adjuvant chemoradiation trial; operable Stage IV oral cancers benefited from adjuvant chemoradiation [26, 27].

The median survival seen in our study is lower when compared to that reported for two-drug and three-drug regimens in the TAX 323 and TAX 324. This difference may be due to fewer oral cavity patients in the above trials [11, 12]. Also, the median follow-up is shorter as compared to TAX 323 and TAX 324. This can be another reason for a lower median survival. Multiple evidence suggests that patients who undergo surgery have a better outcome than those who did not undergo resection in oral cavity cancer [13–15]. Neoadjuvant chemotherapy followed by surgery may improve the overall outcome and lead to an increase in OS. We also observed in our study that a significant number of patients tolerated radical intent nonsurgical therapy, which may be CT RT or RT alone. The outcomes of these patients are also not inferior.

## Conclusion

Our study shows that neoadjuvant chemotherapy in technically unresectable HNC patients can make the disease resectable in around one-third of the patients. The patients who could undergo surgery after neoadjuvant chemotherapy had significantly improved survival as compared to those who could not. The selection of these patients should be done by a multi-disciplinary team and all efforts should be done to make disease resectable to achieve optimal outcomes.

## Conflicts of interest

Nil.

## Funding

The authors did not receive any funding for conducting this study.

## Availability of data and material

The raw data can be made available on appropriate request, while ensuring the anonymity of the study participants.

## Authors’ contributions

The study conception and design was done by BK Mishra. Data collection was performed by BK Mishra, Akhil Kapoor, Anuj Gupta, Bipinesh Sansar, Arpita Singh, Somnath Roy, Tanmoy Mandal, Sujay Srinivas and Sudeep Das. Data analysis was performed by Akhil Kapoor and BK Mishra. The first draft of the manuscript was written by BK Mishra and all authors commented on previous versions of the manuscript. All authors read and approved the final manuscript and guarantee integrity of the entire study.

## Figures and Tables

**Figure 2.**
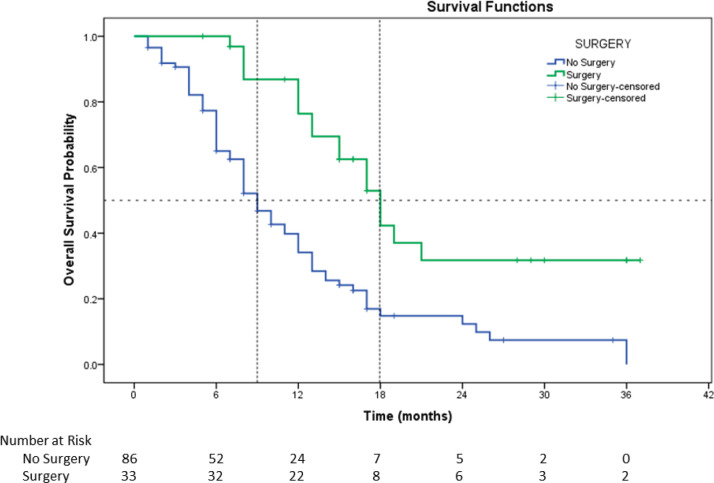
Survival curve of resected patients versus other patients.

**Table 1.** Baseline parameters.

Variable	Patient number
Median age	46 years (23–77 years)
Male/female	Male = 111, Female = 8
Site of cancer (%)	
Oral cavity	104 (87.4)
Laryngopharynx	15 (12.6)
N stage (%)	
N0	23 (19.3)
N1	36 (30.3)
N2	40 (33.6)
N3	20 (16.8)
Reason for neoadjuvant chemotherapy (%)	
Disease reaching up to zygoma and soft tissue swelling up to zygoma	45 (37.8)
Infratemporal fossa involvement	6 (5.0)
Extensive soft tissue up to hyoid and extensive skin infiltration	55 (46.2)
Fix lymph nodes with vascular involvement	15 (12.6)
Regimen	
Three drugs	31 (26.2)
Others	88 (73.8)

**Table 2.** Response rates achieved, resectability at the end of three cycles and toxicity analysis.

Response	Three-drug regimen (*n* = 31) (%)	Two-drug regimens (*n* = 88) (%)
Complete response (CR)	0	0
Partial response (PR)	13 (41.9)	33 (37.5)
Stable disease (SD)	10 (32.3)	35 (39.8)
Progressive disease (PD)	8 (25.8)	20 (22.7)
Resectability achieved	10 (32.3)	23 (26.1)
Resectability not achieved	21 (67.7)	65 (73.9)
**Grade 3–4 toxicity**		
Anaemia	3 (9.7)	23 (26.1)
Febrile neutropenia	6 (19.4)	9 (10.2)
Thrombocytopenia	0	5 (5.7)
Vomiting	1 (3.2)	3 (3.4)
Diarrhoea	3 (9.7)	4 (4.6)
Cardiac event	0	1 (1.1)

**Table 3.** Response as per the indication of NACT.

Indication (n)	PR	SD	PD	Unknown
Disease or oedema up to zygoma (*n* = 43)	21 (48.8%)	9 (20.9%)	13 (30.2%)	0
ITF involvement (*n* = 6)	1 (16.7%)	3 (50%)	1 (16.7%)	1 (16.7%)
Hyoid/skin involvement (*n* = 55)	20 (36.3%)	26 (47.2%)	9 (16.4%)	0
Fix nodes (*n* = 15)	4 (26.7%)	7 (46.7%)	4 (26.7%)	0

PR, Partial response; SD, Stable disease; PD, Progressive disease; ITF, Infratemporal fossa

**Table 4.** Post-neoadjuvant treatment offered.

Modality	Number of patients (%)
SU + CT + RT	30 (25.2)
SU + RT	3 (2.5)
CT + RT	23 (19.3)
Palliative chemotherapy	48 (40.4)
Palliative radiotherapy	8 (6.7)
Supportive care	7 (5.9)

SU, Surgery; CT, Chemotherapy; RT, Radiotherapy
